# Breaking barriers: A decade-long institutional journey to sustain mandated living kidney-donor follow-up in Qatar

**DOI:** 10.5339/qmj.2026.39

**Published:** 2026-06-23

**Authors:** Muhammad Asim, Essa Abdulla Abuhelaiqa, Hassan Ali Al Malki

**Affiliations:** 1 Division of Nephrology, Department of Internal Medicine, Hamad General Hospital, Hamad Medical Corporation, Doha, Qatar

**Keywords:** Living kidney donation, donor follow-up, Qatar transplant program, donor education, coordinator-driven outreach, quality improvement

## Abstract

**Introduction:**

Living kidney donation is essential for expanding transplant opportunities, given the worldwide scarcity of deceased donor organs. Kidney donors experience a slight but detectable long-term increase in risks such as hypertension, diabetes, and end-stage kidney disease, making sustained follow-up essential. However, adherence to post-donation surveillance remains inconsistent worldwide.

**Methods:**

This descriptive evaluation included living kidney donors enrolled in Qatar’s national follow-up program at Hamad Medical Corporation, implemented in January 2017. The program mandates nephrology follow-up at 1 and 6 months post-donation and annually thereafter, with standardized clinical and laboratory monitoring. Adherence rates from 2017 to 2024 were assessed against institutional benchmarks (>90%).

**Results:**

Of 349 registered donors nationwide, 233 donated after program implementation. Despite a fourfold increase in eligible donors between 2017 and 2024, 1- and 6-month follow-up rates remained near 100%, and annual adherence consistently exceeded 90% from 2020 onward. Compliance with the nephrologist-led review of clinical and laboratory data approached 100% among attendees. Sustained performance was temporally associated with system-level interventions, including coordinator-led outreach, structured process redesign, enhanced donor education, and continuous quality improvement initiatives. Follow-up rates also remained robust during the COVID-19 pandemic despite the transition to telephone-based consultations.

**Conclusion:**

A centralized, quality-driven national framework can achieve durable, high-level adherence to long-term follow-up among living kidney donors, even amid rapid program expansion. This scalable model provides a strong foundation for registry-based outcome monitoring and evidence-informed donor care.

## 1. INTRODUCTION

Kidney transplantation provides superior survival and quality of life compared with dialysis.^1^ However, the persistent shortage of deceased donor organs limits access, making living kidney donation an essential alternative. Although the absolute risk of major complications—such as cardiovascular events, end-stage kidney disease (ESKD), and mortality—remains low for living kidney donors, evidence indicates a small but measurable increase in the long-term risk of ESKD and other chronic conditions compared with healthy non-donors.^2–4^ This elevated risk is mainly linked to post-donation hypertension, diabetes, obesity, reduced nephron mass, genetic predisposition, and younger age (which leads to longer lifetime exposure). Notably, hypertension, diabetes, and glomerulonephritis together account for more than 60% of ESKD cases among donors.^5^

Given these potential risks, the transplant community bears a responsibility to ensure rigorous pre-donation evaluation and ongoing post-donation surveillance, particularly for medically complex donors, who are increasingly being considered in response to rising transplant demand.^6^ In the United States, the 2013 OPTN/UNOS policy requires reporting of follow-up data at discharge (or within 6 weeks), and again at 6 months, 1 year, and 2 years after donation. For donations occurring after December 31, 2014, the policy set compliance targets of 80% for clinical data and 70% for laboratory data.^7^ Despite these requirements, a 2017 national report found that only 43% of transplant centers met these benchmarks.^8^ Even government-funded programs have faced persistent challenges in achieving sustained long-term donor follow-up.^9,10^

At Hamad Medical Corporation, Qatar, recommendations for post-donation follow-up of living kidney donors were developed in 2016 and implemented on January 1, 2017. This paper provides a descriptive account of the program’s structural evolution, the challenges encountered, and the strategies adopted to maintain adherence to follow-up protocols over a decade. These outcomes were made possible through the strong commitment of key stakeholders, including the government, the transplant program, and the donors themselves.

## 2. LIVING KIDNEY-DONOR FOLLOW-UP PROGRAM IN QATAR

Qatar has a diverse and dynamic population. With nationals comprising <20% of its 3 million residents, the country has seen a substantial influx of foreign workers—primarily from developing nations—resulting in a multicultural society. Kidney transplantation services are centralized at Hamad General Hospital, located in the capital city of Doha and forming part of the Hamad Medical Corporation network. A government-issued health card provides heavily subsidized access to public healthcare services. The living kidney-donor follow-up program mandates post-donation follow-up at 1 week, 1 month, and 3 months with transplant surgeons, and at 1 month, 6 months, and annually thereafter with transplant nephrologists. For donors residing in Qatar, the program recommends lifelong follow-up. This approach aligns with the expressed wishes of many donors, who value ongoing health surveillance for early detection of complications and to avoid feelings of abandonment—a concern highlighted in a recent study.^11^ For donations performed after December 31, 2016, the program aims to monitor more than 90% of eligible donors, collecting both clinical and laboratory data. Parameters include blood pressure (BP), body mass index (BMI), serum creatinine, fasting blood glucose, urine protein–creatinine (P/C) or albumin–creatinine (A/C) ratio, and urinalysis. The policy is intentionally more rigorous than OPTN/UNOS guidelines for several reasons. First, a considerable proportion of donors return to their home countries soon after donation; therefore, additional early postoperative evaluations are scheduled to ensure proper recovery. Second, evidence indicates that common post-donation morbidities—such as hypertension, diabetes, and proteinuria—often develop more than two years after donation, underscoring the value of extended follow-up.^12^ Third, Qatar’s donor population is ethnically diverse and has a higher baseline prevalence of hypertension, diabetes, obesity, and cardiovascular disease, all of which increase the risk of kidney disease. Long-term outcomes in such high-risk subgroups remain poorly characterized. Finally, nearly one-third of donors are aged 30 years or younger, making prolonged monitoring essential given their extended lifetime exposure to reduced renal reserves.

This study is a descriptive evaluation of longitudinal performance-improvement initiatives designed to sustain mandated living donor follow-up in Qatar, rather than a hypothesis-testing interventional trial. The primary endpoint was adherence to institutional benchmarks (>90%), reflecting operational quality targets rather than comparative clinical outcomes. We report annual adherence rates and temporal trends to evaluate program effectiveness and sustainability over the first decade. Clinical outcome analyses (e.g., hypertension, diabetes, proteinuria, and ESKD) will be presented separately.

Between 1981 and December 31, 2024, a total of 349 kidney donors were registered in the transplant program in Qatar. Although modest compared with large registry studies, this cohort represents the entire national living donor population during the study period rather than a sampled subset. Of these, 233 donations occurred after the implementation of the follow-up guidelines on January 1, 2017. Transplant activity in Qatar before 2017 was very limited because of structural and programmatic constraints. Following the national restructuring of transplant services, centralization of care, expansion of surgical capacity, and increased governmental support, transplant activity increased substantially. Donors who initiated renal replacement therapy (RRT) or died were considered to have exited the follow-up program and were excluded from subsequent adherence analyses. Donors who left Qatar were also excluded, as adherence was calculated only among donors who remained actively eligible for follow-up within the country. This approach reflects program performance in the population for whom monitoring is operationally feasible within a centralized national system. Eight donors residing in Qatar decided not to enroll in the program and instead chose to follow up with their primary nephrologists.

Adherence to 1- and 6-month and annual nephrology follow-up visits was evaluated for the period from January 1, 2017, to December 31, 2024. At each visit, we assessed whether key clinical and laboratory parameters (BP, BMI, serum creatinine, fasting glucose, urine P/C or A/C ratio, and urinalysis) were obtained and reviewed by a nephrologist for those who attended follow-up visits. Implementation of the Cerner electronic health record system in 2015 streamlined data capture and analysis. Documentation of nephrology review was mandatory, data were independently audited annually by an external quality reviewer, and any decline in performance triggered interventions through a continuous quality improvement (CQI) methodology.

## 3. FOLLOW-UP RESULTS

**Figure 1.**
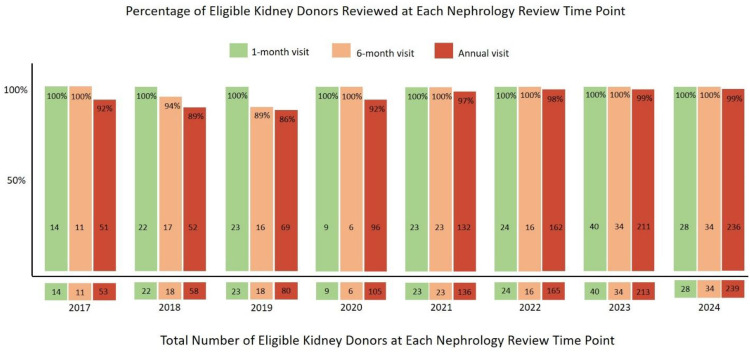
Compliance with the program-advocated follow-up schedule. The boxes below the x-axis show the total number of eligible kidney donors at each nephrology review time-point (1 month, 6 months, and annual follow-up) per year. Numbers within the bars indicate the number of donors reviewed, and the percentages above the bars represent the proportion reviewed (number reviewed ÷ number eligible × 100).

**Figure 2.**
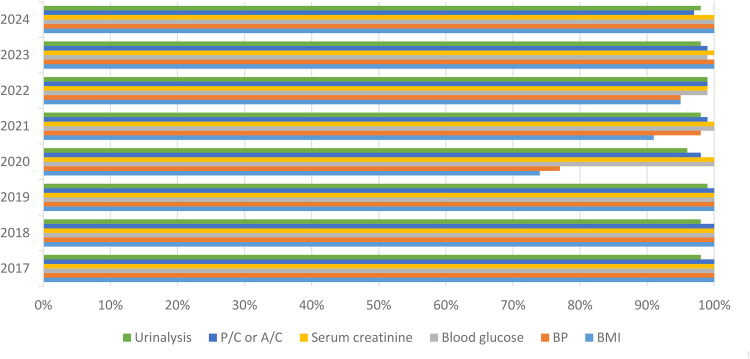
Compliance with program-recommended data review by a nephrologist.

Figure 1 illustrates adherence to postoperative follow-up between January 1, 2017, and December 31, 2024. Follow-up rates remained high despite a fourfold increase in the total number of eligible donors, from 78 in 2017 to 301 in 2024, reflecting the cumulative rise in donations after 2017. The 1- and 6-month follow-up rates remained near 100% throughout 2017–2024, while annual follow-up rates remained above 90%, except for slight declines to 89% and 86% in 2018 and 2019, respectively (Figure 1). Compliance with nephrologist-led review of clinical and laboratory data was nearly 100% among attendees (Figure 2).

During the peak waves of the COVID-19 pandemic in 2020 and 2021, our program transitioned to telephone consultations. Requirements for in-person blood pressure and BMI measurements were temporarily waived, resulting in lower capture rates for these parameters, which were recorded primarily during other healthcare encounters, such as visits to primary care centers. However, laboratory monitoring continued at designated outpatient facilities. Donors were scheduled to complete blood and urine testing prior to their telephone consultations.

Variations in follow-up eligibility reflect the transient and dynamic nature of Qatar’s population. Many expatriate donors return to their home countries soon after donation because of the temporary nature of their employment contracts. In some cases, related donors travel to Qatar specifically for transplantation and return home after recovery. Less commonly, both donor and recipient from low-resource countries travel to Qatar for transplantation and depart after convalescence. Conversely, some Qatari donor–recipient pairs undergo transplantation abroad at partner institutions and remain overseas for extended periods. Since 2020, the centralization of kidney-donor follow-up at the transplant center has also influenced these trends.

## 4. DISCUSSION

Prior to 2017, the kidney-donor clinic primarily focused on pre-donation evaluation, with limited infrastructure for structured post-donation follow-up. Data collection was confined to the initial hospitalization period, and subsequent follow-up was irregular and poorly coordinated, resulting in incomplete clinical and longitudinal data. Clinic scheduling was managed by general nephrology clerks rather than dedicated transplant coordinators, and donors were typically reviewed only if they developed comorbidities. Consequently, missed appointments frequently led to donors being lost to follow-up.

Concurrent with the 2017 implementation of institutional recommendations for kidney-donor follow-up, we addressed key barriers to long-term donor monitoring—donor inconvenience and the financial burden of ongoing care, despite most costs being incurred perioperatively^8,13–15^ (Table 1). Our program provided free healthcare to all donors, streamlined scheduling to reduce waiting times, and ensured consistent, multilingual care teams to improve communication. Early education emphasized the importance of lifelong follow-up. Two full-time transplant coordinators—registered nurses with transplant program experience—oversaw scheduling, maintained updated contact information, and conducted proactive reminders. Reporting directly to the Director of Transplantation, they worked without assistants.

Initial outcomes were encouraging, with 1-month, 6-month, and annual follow-up rates exceeding 90%. However, as donor numbers increased between 2017 and 2019, some donors—particularly asymptomatic individuals—remained reluctant to attend long-term follow-up, with annual adherence declining to 86%, while 1- and 6-month follow-up rates remained high.

**Figure 3.**
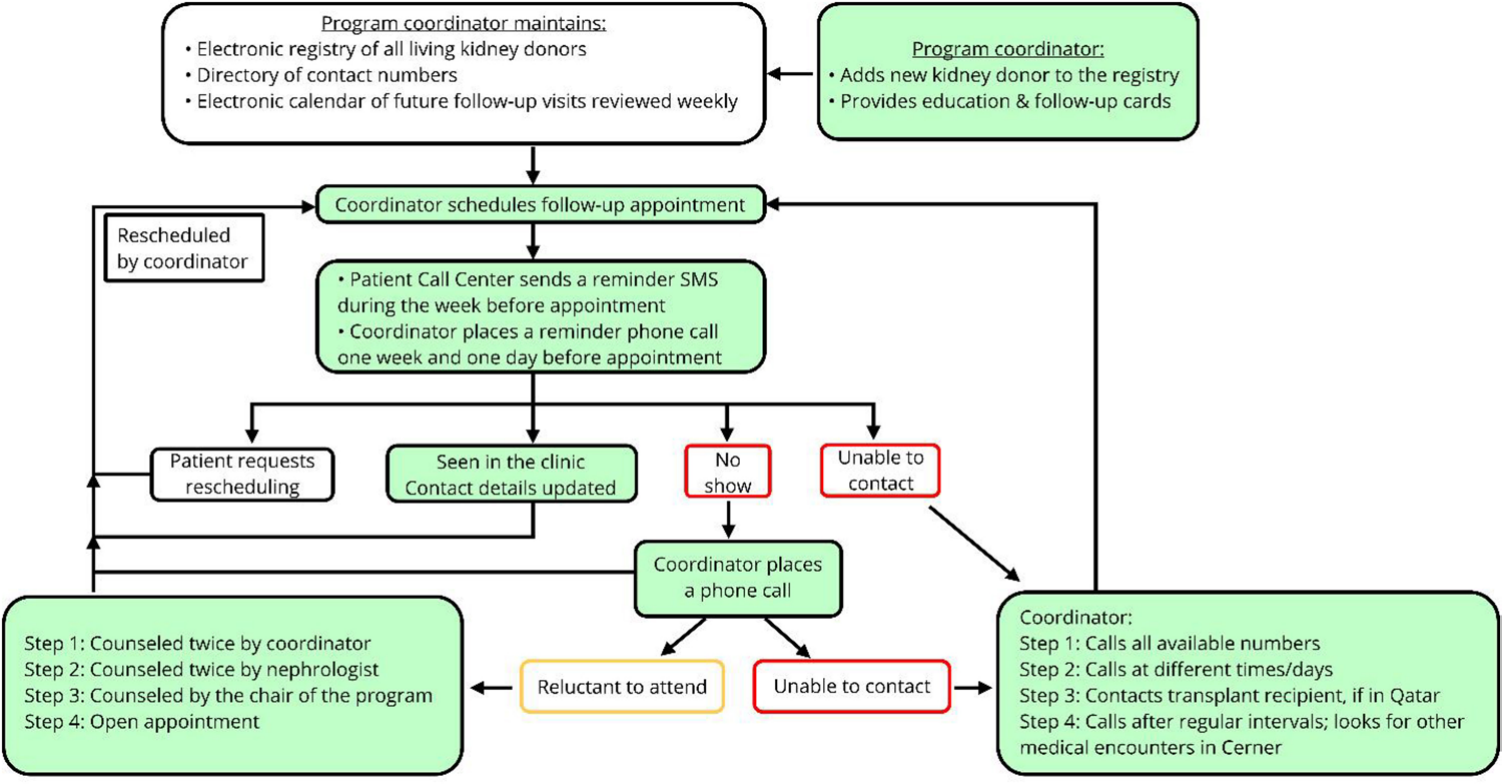
Process map for managing follow-up procedures, targeting no-shows, unreachable individuals, and reluctant attendees.

In response, a second set of interventions was introduced in 2020, including the development of a process map to standardize follow-up procedures and address barriers related to attendance, donor reluctance, and accessibility (Table 1 and Figure 3). Following these measures, 1- and 6-month adherence reached 100%, and annual adherence consistently exceeded 90%, despite a fourfold increase in donors eligible for annual review compared with 2017. Compliance with the nephrologist-led review of clinical and laboratory data approached 100% among attendees. Follow-up rates did not differ among younger donors—who constitute approximately one-third of the donor pool and are considered a high-risk phenotype due to a greater likelihood of missing scheduled follow-up (which hinders effective monitoring of their health outcomes).^16^

During the COVID-19 pandemic, the OPTN temporarily suspended follow-up reporting requirements (effective April 3, 2020). In response, our program shifted to telephone consultations during the 2020–2021 pandemic peaks in Qatar, with laboratory monitoring maintained at designated outpatient facilities prior to each call. Notably, follow-up rates remained above 90% at all time points, highlighting system resilience.

Coordinators rescheduled 40% of visits, likely preventing missed appointments through proactive outreach. In contrast, no-show rates among general nephrology patients attending general nephrology clinics—typically around 20%—rose to approximately 40% during the pandemic, despite the availability of telephone-based virtual visits.

Further interventions were implemented in 2022 and 2023 to address the challenges associated with the expanding donor population (Table 1). These included the introduction of the Donor Follow-Up Tracker, an Excel-based calendar alert system designed to help coordinators manage upcoming appointments and reduce scheduling lapses. To address occasional deviations from the process map, coordinators were required to document key measures, such as rescheduling rates, directly in the Cerner electronic health record. Educational tools, including wallet cards and informational leaflets, were introduced to emphasize the donor’s responsibility in maintaining their own health. The institution also launched patient portals to enhance self-monitoring and communication. These measures reduced rescheduling rates (for no-shows) from 22% in 2022 to 18% in 2023 and 11% in 2024, reflecting improved donor engagement and compliance.

## 5. INTERNATIONAL COMPARISONS

Living kidney-donor follow-up rates vary considerably across countries, influenced by national policies, healthcare infrastructure, donor engagement strategies, and donor population characteristics. International evidence consistently shows a decline in follow-up attendance and laboratory data capture over time. In the United States, despite the 2013 OPTN/UNOS policy mandating donor follow-up at 6 months, 1 year, and 2 years, a 2017 national registry analysis found that only 43% of transplant centers met these requirements for donations performed in 2013.^8^ Another large U.S. study reported significantly lower follow-up rates among international donors compared with domestic donors at 6, 12, and 24 months post-nephrectomy (45%, 33%, and 36% vs. 76%, 71%, and 70%, respectively).^17^ In Japan, a single-center study found follow-up rates of 83.9% at 1 year, 74.6% at 2 years, and 59.2% at 5 years, with 42% of donors lost to follow-up. The authors highlighted the urgent need for a national registry to support long-term donor monitoring.^18^ In Canada, recent data from Alberta demonstrated that early, guideline-concordant care substantially improved long-term follow-up. Donors without early follow-up had 76% lower odds of annual review at 5 years and 68% lower odds at 10 years.^19^ An Australia and New Zealand Living Kidney Donor analysis (2003–2021) reported follow-up rates of approximately 38–49% at 1 year and 28–35% at 2 years (varying by donation era), with further decline beyond 2 years.^20^ Some international programs have introduced reimbursement policies, although these often include caps on the total amount donors can claim.

## 6. LIMITATIONS

Our study has several limitations. First, it was conducted within Qatar’s centralized, government-funded healthcare system, where donor care is provided free of charge, which may limit generalizability to other settings. However, key program elements—including coordinator-led outreach, structured process mapping, reinforced education, and CQI methodology—are transferable across different healthcare models. Second, formal cost-effectiveness analyses were not performed, although the program used existing transplant infrastructure and coordinator roles, thereby minimizing additional costs. Third, quantitative adherence data before 2017 were not systematically recorded, precluding direct pre–post comparisons; therefore, findings were contextualized using international benchmarks and internal targets. Fourth, individual intervention effects could not be isolated because components were implemented in bundled phases. While causality cannot be established, the temporal association between structured process redesign and sustained improvements in follow-up rates suggests that system-level quality initiatives are feasible and potentially effective. However, these improvements may also reflect unmeasured factors, such as broader institutional changes or increased societal awareness, which cannot be fully disentangled. Finally, eight donors residing in Qatar chose to pursue follow-up outside the centralized national program and were therefore excluded from adherence analyses. Although this represents a small proportion of the cohort (<3%) and is unlikely to have materially influenced adherence estimates, their exclusion may introduce minimal selection bias.

## 7. FUTURE DIRECTIONS

As the donor population continues to expand and lifelong follow-up remains resource-intensive, we are exploring a personalized, risk-stratified model of care. Under this approach, high-risk donors—such as those who are medically complex, first-degree relatives of recipients, or those who develop comorbidities—could continue with annual reviews. In contrast, donors with a stable health status could transition to biennial follow-up after the first two years. Norway’s structured follow-up model, which schedules annual visits for the first five years followed by reviews every five years thereafter, has achieved robust long-term adherence.^21^ Alternatively, stable donors could be monitored by primary care providers, with clear pathways for re-referral to the transplant center if concerns arise. Such a stratified framework would optimize resource usage while preserving care quality.

Currently, donor data are maintained in a retrospective Excel-based database that functions as a basic registry but lacks standardized definitions and uniform data entry procedures. To strengthen long-term monitoring, we are developing a National Registry to enable standardized data capture, outcome tracking, and international collaboration. Evidence suggests that formal registries, combined with standardized care pathways and well-organized follow-up systems, improve donor adherence rates and guide evidence-based improvements in living donor evaluation and follow-up protocols.^18,22^ Once implemented, the registry will support centralized oversight and ensure continuity of care, even when donors transition to other centers—an important advancement now that a strong culture of donor follow-up has been firmly established.

At present, there are no published data on the long-term incidence of ESKD, hypertension, diabetes, or other complications among living kidney donors in Qatar. Outcome data generated through our emerging national donor cohort will be instrumental in identifying donor health trends, informing clinical guidelines, and refining informed consent processes. This highlights the broader significance of structured follow-up programs and the potential for our findings to guide both clinical practice and national donor-care policy.

## 8. CONCLUSION

In Qatar, the past decade has seen the transformation of the donor follow-up program from a fragmented and inconsistent process into a structured, high-compliance program. This progress reflects the collective commitment of all stakeholders, including Hamad Medical Corporation, the kidney transplant program, and, importantly, the kidney donors themselves. Key drivers of success have included cost-free follow-up care, personalized education, targeted processes to address no-shows and disengagement, and an embedded culture of CQI. Together, these measures have produced sustained gains: since 2020, 1- and 6-month follow-up compliance has remained at 100%, and annual follow-up rates have consistently exceeded 90%, despite a fourfold increase in donor volume since 2017. This scalable model provides a foundation for registry-based outcome monitoring and evidence-informed donor care. Our experience shows that sustained donor follow-up is both feasible and scalable. We encourage transplant programs worldwide to regard long-term donor care not merely as a regulatory obligation, but also as a clinical priority and an ethical responsibility. 

## ACKNOWLEDGMENT

We are grateful to Nicoleta Stanciu and Sabera Baqi, the program coordinators, and to Sahar Aly, the QI reviewer, for their support and commitment to the program.

## COMPETING INTERESTS

The author(s) declare that there is no conflict of interest.

## AUTHORS’ CONTRIBUTIONS

All authors have contributed to the conceptualization, writing, and review of the manuscript.

## FUNDING

The author(s) received no financial support for the research, authorship, and/or publication of this article.

## DISCLOSURE OF AI USE

AI-assisted tools were used solely for grammar and language editing.

## Figures and Tables

**Table 1.** Strategies that enabled guideline-concordant follow-up.

Interventions	Description

Providing free access to healthcare	Offered free healthcare for kidney donors in Qatar, including consultations, laboratory tests, and medications
Minimizing donor inconvenience	Streamlined clinic schedules (AM/PM); allocated dedicated time slots to reduce waiting times
Building strong donor relationships	Established continuity of care by assigning the same nephrologists and coordinators; improved communication through multilingual staff
Educating the kidney donors	Emphasized post-donation follow-up during registration to enable early detection and management of comorbidities
Preventing loss to follow-up	Managed clinic scheduling through program coordinators; verified donor and next-of-kin contact details regularly; conducted personal outreach through reminders and phone calls by coordinators up to one week in advance to confirm laboratory and clinic attendance

Refining process map	Redesigned follow-up workflows to address no-shows, reluctant attendees, and unreachable donors (Figure 3)
Using CQI methods	Implemented CQI techniques to monitor, review, and revise organizational procedures when follow-up benchmarks were not met

Addressing occasional missed scheduling by coordinators	Launched the Donor Follow-Up Tracker—an Excel spreadsheet calendar that consolidates each donor’s contact details, past visits, and upcoming appointments —reviewed weekly by coordinators
Tracking process measures	Mandated Cerner documentation by coordinators to track process measures for rescheduling, no-shows, and contact losses
Intensifying donor education initiatives	Ensured comprehension of the follow-up plan prior to surgery, thereby improving the informed consent of prospective donors; introduced post-donation care plans signed by donors; distributed educational leaflets and wallet-sized reminder cards
